# Phenotype in Individuals with Heterozygous Rare Variants in *LIPC* Encoding Hepatic Lipase

**DOI:** 10.3390/ijms252111445

**Published:** 2024-10-24

**Authors:** Erin O. Jacob, Jian Wang, Adam D. McIntyre, Robert A. Hegele

**Affiliations:** 1 Robarts Research Institute, Schulich School of Medicine and Dentistry, Western University, 4288A-1151 Richmond Street, London, ON N6A 5B7, Canadajwang@robarts.ca (J.W.); amcintyre@robarts.ca (A.D.M.); 2 Department of Biochemistry, Schulich School of Medicine and Dentistry, Western University, 1151 Richmond Street, London, ON N6A 3K7, Canada; 3 Department of Medicine, Schulich School of Medicine and Dentistry, Western University, 1151 Richmond Street, London, ON N6A 3K7, Canada

**Keywords:** hepatic lipase deficiency, hepatic lipase, recessive, LIPC gene variant, dyslipidemia, hypercholesterolemia, next-generation DNA sequencing, pathogenic variant, bioinformatics

## Abstract

Hepatic lipase deficiency is a rare genetic condition caused by biallelic loss-of-function variants in the *LIPC* gene encoding hepatic lipase. These variants reduce or abolish the protein’s lipolytic activity, resulting in elevated plasma lipids. The condition is classified as autosomal recessive, since dyslipidemia is inconsistently observed in heterozygous patients with only one *LIPC* variant. However, this has been concluded from historical studies encompassing a few families and having very small sample sizes. Here, we conduct a retrospective chart review of 46 heterozygous subjects, each harboring one rare pathogenic *LIPC* variant. We compare plasma levels of total cholesterol, low-density lipoprotein cholesterol (LDL-C), high-density lipoprotein cholesterol (HDL-C), triglycerides, and apolipoprotein B to those of matched controls without *LIPC* variants. Variant pathogenicity is classified according to the guidelines of the American College of Medical Genetics and Genomics. We observe that levels of total cholesterol, LDL-C, and triglycerides are significantly elevated in the *LIPC* variant heterozygotes, but HDL-C and apolipoprotein B are not. When filtering solely with respect to pathogenic or likely pathogenic variants, all lipid variables emerge as significantly elevated compared to controls. Thus, hepatic lipase deficiency may not necessarily be a recessive condition, but perhaps semi-dominant since individuals with one variant are phenotypically distinct from the controls. These hypothesis-generating findings regarding *LIPC* genetic variations observed in a clinical cohort should be evaluated in larger populations and databases.

## 1. Introduction

Hepatic lipase (HL) is a protein secreted by the liver that is involved in lipid metabolism, encoded by the *LIPC* gene on chromosome 15q21.3 [1]. HL hydrolyzes triglycerides (TG) and phospholipids from circulating lipoproteins [1]. The most significant role of HL is the conversion of intermediate-density lipoprotein (IDL) to low-density lipoprotein (LDL) [1]. HL also facilitates high-density lipoprotein (HDL) metabolism and has a non-catalytic function to promote hepatic LDL uptake by the LDL receptor-related protein 1 (LRP1) [1,2]. Defective function of HL may lead to dyslipidemia and atherosclerosis [3].

HL deficiency is a very rare genetic condition that occurs when biallelic loss-of-function variants in *LIPC* either reduce or abolish lipolytic function [1]. Fewer than 10 families have been reported in the literature as suffering from HL deficiency, and, thus, HL deficiency has traditionally been presumed to be a very rare condition, although population prevalence has been challenging to estimate accurately and may actually prove to be higher than previously suspected. It is clinically characterized by elevated levels of total cholesterol (TC) and TG and by moderate levels of LDL and HDL cholesterol (LDL-C; HDL-C, respectively) [1,4,5]. LDL and HDL particles are also often abnormally phospholipid- and triglyceride- enriched [6]. Some studies have demonstrated a link between HL deficiency and atherosclerosis; however, larger-scale population studies must be conducted to confirm this [7,8,9]. This could be a challenge, as it is a rare condition and, therefore, difficult to evaluate [7].

HL deficiency is currently recognized as a recessive condition, as simple heterozygotes have not been consistently observed to display dyslipidemias [7]. However, most of these studies were conducted in the 1990s, when DNA sequencing was less accessible. Conclusions about heterozygosity for HL deficiency from that era were based on family studies with very small sample sizes, where detecting mild-to-moderate phenotypic trends would be challenging [7,8]. Furthermore, it was proposed that a second factor would be necessary for expression of dyslipidemia in simple heterozygotes for *LIPC* variants [8,10]. For example, mice with concurrent loss-of-function variants in both the *LIPC* and *LDLR* genes have been found to exhibit more extreme dyslipidemia and atherosclerosis compared to those harboring either variant alone [11]. This is because the *LDLR* gene encodes the LDL receptor, the variants of which can cause familial hypercholesterolemia (FH) [12]; such findings suggest a potential interaction with *LIPC* variants.

In order to revisit the question of the phenotype in simple heterozygotes for *LIPC* variants, we performed a retrospective chart review of genetically defined lipid disorders in clinic patients. Our findings suggest that simple heterozygotes for *LIPC* variants have detectable dyslipidemia phenotypes, and that hepatic lipase deficiency may not be a purely recessive condition.

## 2. Results

### 2.1. Study Subjects

The procedure used to filter subjects based on the inclusion criteria is shown in the Supplementary Figure S1. Of the 54 subjects with rare *LIPC* variants, 53 were simple heterozygotes, and 46 did not harbor additional pathogenic variants associated with FH in either the *LDLR*, *APOB*, *PCSK9*, or *LDLRAP1* genes. None of the subjects had pathogenic variants associated with hypertriglyceridemia.

### 2.2. Demographic and Genomic Information

Clinical and demographic information on the *LIPC* variant and control groups are outlined in Table 1. Two patients with *LIPC* variants had elevated ALT levels consistent with fatty liver disease. The control cohort comprised 72 subjects with normal lipid levels, no rare variants identified by the LipidSeq targeted DNA sequencing panel, and who were, otherwise, healthy. Information about the *LIPC* variants present in the cohort are outlined in the Supplementary Table S1. One novel variant, p.His46Pro (H46P), was identified. Levels of total (TC) and low-density lipoprotein (LDL) cholesterol, as well as triglycerides (TG), were significantly higher in simple heterozygotes with *LIPC* variants compared to controls (all *p* < 0.05).

### 2.3. Association Between LIPC Variants and Lipid Levels

**Figure 1:**
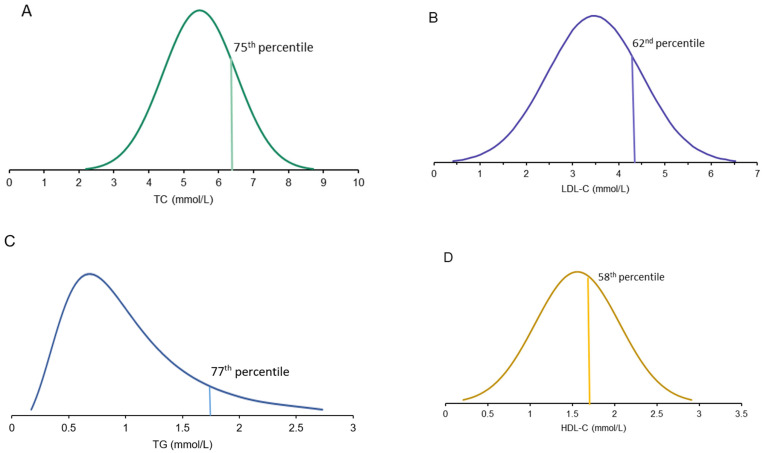
Lipid and lipoprotein levels of patients with *LIPC* variants compared to population controls. The plasma levels of total cholesterol (TC) (**A**), low-density lipoprotein cholesterol (LDL-C) (**B**), high-density lipoprotein cholesterol (HDL-C) (**C**), and triglycerides (TG) (**D**) were measured in patients with *LIPC* variants. Each patient’s levels were converted to percentile values, with the average percentile of the cohort displayed above (vertical line). The reference percentile values were obtained from the LRC prevalence study (North America). The distributions were formed using data from 50–55-year-old women, corresponding to the average age and sex of the cohort.

**Figure 2:**
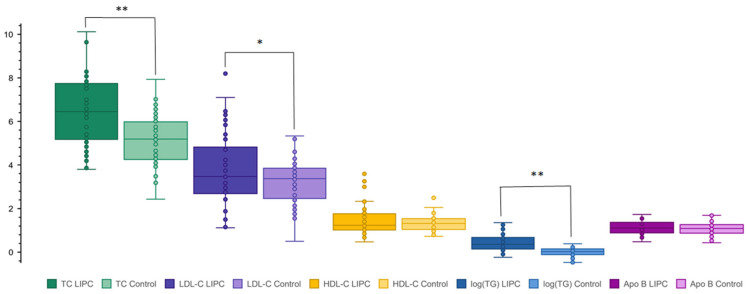
Lipid levels of patients with *LIPC* variants compared to normal controls. Plasma levels of total cholesterol (TC) (green), low-density lipoprotein cholesterol (LDL-C) (purple), high-density lipoprotein cholesterol (HDL-C) (orange), triglycerides (log(TG)) (blue), and apolipoprotein (apo) B (pink) were compared between patients with *LIPC* variants (*n* = 46) and normal controls (*n* = 72). The darker color corresponds to the *LIPC* cohort, the lighter to the control. Unpaired one-tailed *t*-tests were used to compare the lipid levels between cohorts: ‘*’ denotes a *p*-value < 0.05 and ‘**’ denotes a *p*-value < 0.0001. The one-tailed *p*-value between *LIPC* and control groups is 1.5 × 10^−6^ for TC, 0.019 for LDL-C, 0.11 for HDL-C, 5.4 × 10^−9^ for log(TG), and 0.20 for Apo B.

Next, mean levels of TC, LDL-C, high-density lipoprotein (HDL) cholesterol, and TG of simple heterozygotes with *LIPC* variants were compared to the general North American population. The distribution of each variable was obtained from the LRC prevalence study and reported as percentile values corresponding to numerical lipid levels [13]. Each subject’s lipid level was converted to an age- and sex-specific percentile value; the mean percentiles for *LIPC* variant heterozygotes for each variable are shown in Figure 1. TC and TG values showed the greatest deviation from North American median values, at the 75th and 77th percentile, respectively. Less extreme deviations were observed for LDL-C at the 62nd percentile, while HDL-C levels exhibited deviations at the 58th percentile. When comparing the *LIPC* cohort to the group of genetically normal controls (*n* = 72), elevated levels were identified in relation to TC, LDL-C, and TG only: a similar phenotypic pattern to that emerging from previous analyses (Figure 2). Mean lipid and lipoprotein levels and percentile values of cases and controls are summarized in Table 1.

### 2.4. Association Between Variant Pathogenicity and Lipid Levels

**Figure 3:**
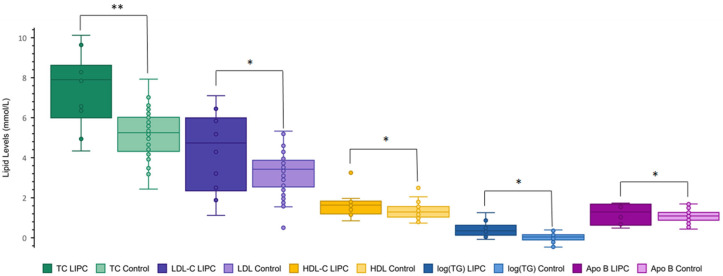
Lipid levels of patients with pathogenic *LIPC* variants compared to normal controls. Plasma levels of total cholesterol (TC) (green), low-density lipoprotein cholesterol (LDL-C) (purple), high-density lipoprotein cholesterol (HDL-C) (orange), triglycerides (log(TG)) (blue), and apolipoprotein B (Apo B) (pink) were compared among patients with pathogenic or likely pathogenic *LIPC* variants (*n* = 13) and normal controls (*n* = 72). The darker color corresponds to the *LIPC* cohort, the lighter to the control. Unpaired one-tailed *t*-tests were used to compare the lipid levels between cohorts, ‘*’ denotes a *p*-value < 0.05 and ‘**’ denotes a *p*-value < 0.0001: TC *p* = 5.7 × 10^−5^, LDL-C *p* = 0.011, HDL-C *p* = 0.039, log(TG) *p* = 5.9 × 10^−3^, and Apo B *p* = 0.005.

**Figure 4:**
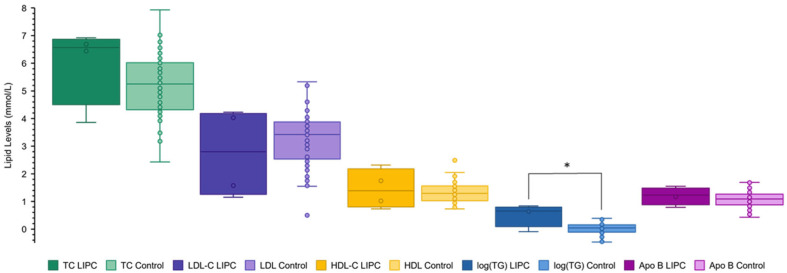
Lipid levels of patients with benign *LIPC* variants compared to normal controls. Plasma levels of total cholesterol (TC) (green), low-density lipoprotein cholesterol (LDL-C) (purple), high-density lipoprotein cholesterol (HDL-C) (orange), triglycerides (log(TG)) (blue), and apolipoprotein B (Apo B) (pink) were compared among patients with benign or likely benign *LIPC* variants (*n* = 4) and normal controls (*n* = 72). The darker color corresponds to the *LIPC* cohort, the lighter to the control. Unpaired one-tailed *t*-tests were used to compare the lipid levels between cohorts, ‘*’ denotes a *p*-value < 0.05. The one-tailed *p*-value between the *LIPC* group and the control group is 0.159 for TC, 0.313 for LDL-C, 0.385 for HDL-C, 0.048 for log(TG), and 0.25 for Apo B.

To assess whether variant pathogenicity influences the observed trends in lipid phenotypes, each variant was classified as ‘pathogenic’ (*n* = 13), ‘benign’ (*n* = 4), or as ‘variants of uncertain significance’ (VUS) (*n* = 29) using the criteria outlined in the ACMG guidelines and the Franklin variable, which was observed to be elevated (Figure 3 and Figure 4, and Table 2).

**Figure 5:**
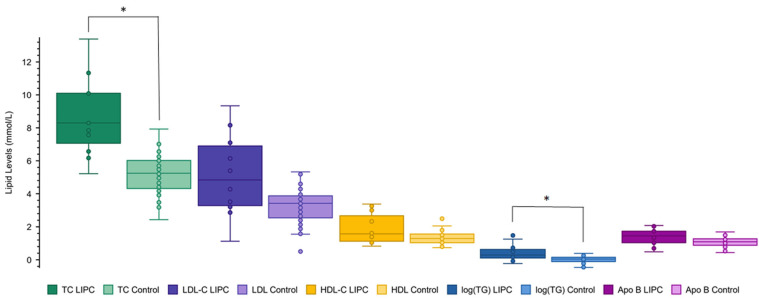
Lipid levels of patients with AI-predicted pathogenic *LIPC* variants compared to normal controls. Plasma levels of total cholesterol (TC) (green), low-density lipoprotein cholesterol (LDL-C) (purple), high-density lipoprotein cholesterol (HDL-C) (orange), triglycerides (log(TG)) (blue), and apolipoprotein B (Apo B) (pink) were compared among patients with pathogenic or likely pathogenic *LIPC* variants (*n* = 10) and normal controls (*n* = 72). The pathogenic classification was obtained using the AI predictive software VARITY (v1.0). All samples scored 0.823 or higher, with scores closer to 1.00 indicating a higher likelihood of pathogenicity. The darker color corresponds to the *LIPC* cohort, the lighter to the control. Unpaired one-tailed *t*-tests were used to compare the lipid levels between cohorts, ‘*’ denotes a *p*-value < 0.05: TC *p* = 1.9 × 10^−3^, LDL-C *p* = 0.061, HDL-C *p* = 0.075, log(TG) *p* = 6.0 × 10^−3^, and Apo B *p* = 0.31.

The pathogenic group comprised 13 patients with pathogenic or likely pathogenic variants, while the benign group consisted of four patients with benign or likely benign variants. The VARITY group contained 10 subjects with variants predicted to be pathogenic by the AI algorithm. The *p*-values from one-tailed unpaired *t*-tests are reported, comparing each group to the control cohort. The VARITY AI prediction algorithm was used as an alternative method of assessing pathogenicity. A total of 21/26 of the variants present in the cohort had a VARITY score available for analysis, encompassing 37/46 total subjects (Supplementary Table S1). Of these variants, six were predicted to be pathogenic. Lipid levels of the VARITY-predicted pathogenic group (*n* = 10) were compared to normal controls (*n* = 72) (Figure 5). TC and TG levels were elevated compared to the controls, whereas no significant differences in LDL-C, HDL-C, and Apo B were identified (Table 2).

### 2.5. Assessing the Impact of Co-Occurring LIPC and FH Variants on Lipid Phenotype

**Figure 6:**
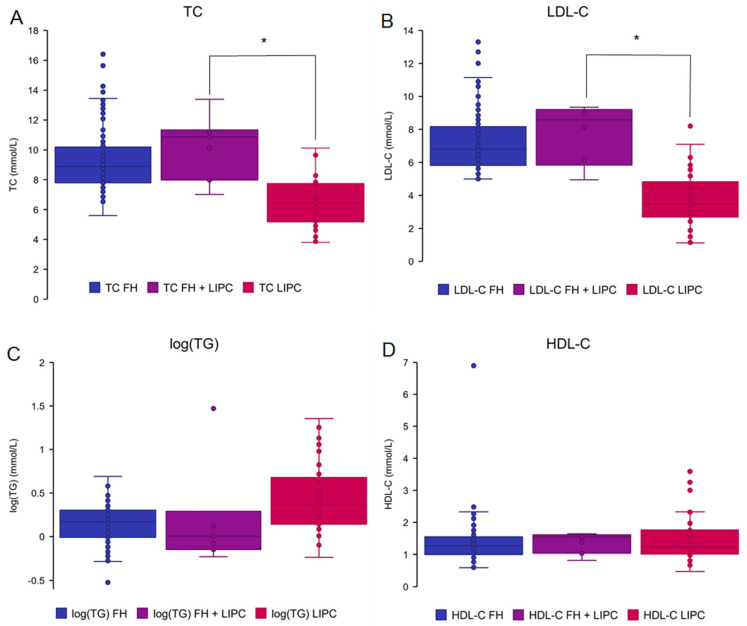
Lipid levels of patients with *LIPC* and familial hypercholesterolemia variants. Plasma lipid levels were compared among patients with both *LIPC* and familial hypercholesterolemia (FH) variants (*n* = 7) (purple) and patients with only *LIPC* variants (*n* = 46) (pink) or only FH variants (*n* = 135) (blue). (**A**) Total cholesterol (TC). The *p*-value between the *LIPC* and FH + *LIPC* groups is *p* = 0.003. (**B**) Low-density lipoprotein cholesterol (LDL-C). The one-tailed *p*-value between *LIPC* and FH + *LIPC* is *p* = 0.002. (**C**) High-density lipoprotein cholesterol (HDL-C). (**D**) Log(triglycerides) (log(TG)). ‘*’ denotes a *p*-value < 0.05. All other comparisons had *p*-values greater than 0.1.

To determine whether lipid elevations are more extreme in simple heterozygotes with concurrent *LIPC* and FH variants, subjects with one *LIPC* and one FH variant were identified (*n* = 7) and their lipid levels compared to subjects with either an *LIPC* variant (*n* = 46) or an FH variant (*n* = 135) (Figure 6). Demographic characteristics and lipid levels of the FH cohort are found in Table 3. There were no significant differences in lipid levels between the FH-only and *LIPC* + FH groups (TC = 10.3 mmol/L, *p* = 0.26; LDL-C = 7.79 mmol/L, *p* = 0.48; HDL-C = 1.37 mmol/L, *p* = 0.84; TG = 5.13 mmol/L, log(TG) *p* = 0.83). Compared to the *LIPC*-only group, the FH + *LIPC* group had elevated TC (*p* = 0.0028) and LDL-C (*p* = 0.0021) levels, characteristic of the FH phenotype. No elevations in TG (log(TG) *p* = 0.36) or HDL-C (*p* = 0.51) levels were observed. Apo B levels were not available for the FH-only cohort and, as such, were not included in this analysis.

## 3. Discussion

The principal findings from this observational study are that individuals who are heterozygous for rare variants in *LIPC* have higher levels of total and LDL cholesterol, as well as TG compared to matched genotypically normal controls. Furthermore, when stringency for pathogenicity of *LIPC* variants was increased, levels of HDL-C and Apo B also became significantly elevated. Our results contrast with those from previous studies, which imply that there was no phenotypic disturbance in simple heterozygotes with *LIPC* variants [7,8]. Our study analyzes the largest cohort of simple heterozygotes to date, reporting data from 46 patients, considerably larger than past sample sizes of three and eight [7,8]. If our findings can be confirmed in larger independent cohorts and datasets, they would suggest that hepatic lipase deficiency might not be a purely recessive condition.

The phenotype of individuals with biallelic pathogenic variants in *LIPC* is a complex combined dyslipidemia with marked elevations in total, LDL, and HDL cholesterol, triglycerides, and Apo B as well as the presence of beta-migrating very-low-density lipoprotein (VLDL), which can be considered functionally equivalent to intermediate density lipoprotein (IDL), as well as abnormal TG enrichment of LDL and HDL species [7,10]. These findings are consistent with the presumed function of HL, which is to further hydrolyze lipolytic remnants of VLDL and also, possibly, the small quantities of TG residing within LDL and HDL [10]. Homozygotes with HL deficiency have pronounced dyslipidemia with presence of VLDL remnant particles (IDL), plus elevated levels of LDL-C and HDL-C along with abnormal TG enrichment of both LDL and HDL [10]. The findings here among heterozygotes suggest that some of the same perturbations in the lipid profile are present directionally, although to a much milder degree than among individuals with biallelic pathogenic variants. Furthermore, we did not have the ability to assess qualitative differences in lipid composition of lipoprotein species or to detect the abnormal presence of species such as IDL.

Our findings suggest that one pathogenic variant may be sufficient to mildly disrupt metabolism of LDL and TG-rich lipoproteins (VLDL), but two pathogenic alleles are required for more pronounced effects on these species and on HDL. Furthermore, the findings would be consistent with a semi-dominant inheritance pattern of *LIPC* variants with respect to TC, LDL-C, and TG, but recessive with respect to HDL-C. Such an inheritance pattern has been observed in familial combined hypolipidemia caused by disabling *ANGPTL3* variants [14]. In those families, when both copies of *ANGPTL3* harbor deleterious variants, TC, TG, LDL-C, and HDL-C levels are depressed, but, with heterozygosity, HDL-C is normal and the other traits are mildly depressed [14]. Hepatic lipase deficiency could be expressed in a similar way, potentially due to compensatory effects of other factors involved in HDL metabolism [15]. However, when our analysis was restricted to individuals with pathogenic or likely pathogenic variants, HDL-C levels were also observed to be elevated (Figure 3). When only benign or likely benign variants were analyzed, no significant differences were measured, except for TG (log(TG) *p* = 0.048) (Figure 3). Therefore, it is possible that simple heterozygotes harboring the most stringently defined deleterious variants may display elevated levels across all variables.

Therefore, these results provide a starting point for further investigation that characterizes the phenotype of simple heterozygotes in larger datasets with better quality data compared to past literature.

Limitations of the present study include the possibility of additional unmeasured causative variants for dyslipidemia. Although variants in genes causing FH and familial chylomicronemia syndrome were controlled for, potential unmeasured variants in other genes affecting lipoprotein phenotypes might have been present [12,16]. Another potential limitation is the small size of the control group. Age, sex, and body mass index were similar between cohorts, although age-related effects on lipoprotein variables were likely not important here [17], e.g., the impact of age was accounted for in the comparisons with North American population controls. Sample size is also a potential limitation, e.g., only four patients had benign or likely benign variants. Another potential limitation is the classification of variants: 29/46 subjects had variants classified as VUS, since most variants had insufficient information to definitively attribute their pathogenicity. Although the VARITY prediction algorithm was used [18,19], such artificial-intelligence-based methods are not infallible when compared with functional mechanistic experiments. Future studies should evaluate these uncertain variants and classify their impact.

In summary, our observations suggest that heterozygosity for rare pathogenic variants in *LIPC* is associated with perturbations in the lipid profile. While hepatic lipase deficiency has been previously considered to be a recessive condition, our findings suggest a semi-dominant pattern involving multiple lipid and lipoprotein phenotypes including TC, LDL-C, TG, and, possibly, HDL-C. These findings extend our understanding of the impact of hepatic lipase and can serve as a starting point for future investigations in independent, large and diverse populations to determine the degree of risk associated with these variants, ultimately aiming to predict and prevent atherosclerotic cardiovascular disease.

## 4. Materials and Methods

### 4.1. Study Subjects

A retrospective chart review of patients referred to the Lipid Genetics Clinic at the London Health Sciences Center (LHSC), University Hospital, London, Ontario, Canada, was conducted. To be eligible, each subject must harbor one *LIPC* variant, must not harbor variants associated with FH or familial hypertriglyceridemia, and must have lipid values available. Control individuals were selected from family members (unrelated spouses and unaffected relatives) who participated in the screening process and were matched to cases based on sex and age with a 10-year margin. All patients provided informed consent, and the project was approved by the Western University Research Ethics Board (project number 0379).

### 4.2. Biochemical, Clinical, and Demographic Information

Clinical and demographic information was collected during the initial visit to the Lipid Genetics Clinic. Lipid levels were measured from fasting blood samples using routine protocols at the Core Biochemistry Laboratory at LHSC, University Hospital [20]. Lipid profiling included determinations of TC, LDL-C, HDL-C, TG, and apolipoprotein (Apo) B. TC, TG, and HDL-C were measured using enzymatic colorimetric analysis. Apo B was measured using an enhanced immunoturbidometric assay. LDL-C levels in subjects with TG values lower than 4.5 mmol/L were determined using the Friedewald formula (i.e., LDL-C = TC minus HDL-C minus TG/2.2); those with TG values between 4.5 mmol/L and 9 mmol/L were calculated using a formula developed by Sampson et al. [21].

The percentile score of each patient’s lipid values based on their age and sex was also recorded and was used for comparisons. Reference percentile values were obtained from North American population data from the Lipid Research Clinics (LRC) prevalence study [13]. Excel was used to extrapolate these data, using a quadratic function to obtain a percentile score for every possible lipid value, specific to sex and age.

### 4.3. DNA Sequencing and Variant Identification

DNA from whole-blood samples was extracted and sequenced at the London Regional Genomics Centre using established methods [22]. Genetic variants were identified using the next-generation DNA sequencing panel LipidSeq using previously described methods [22]. LipidSeq is a targeted exon sequencing panel run on the Illumina MiSeq DNA sequencer that identifies variants present in 69 genes linked to altered lipid metabolism and dyslipidemia, including *LIPC* [22]. Patients with rare *LIPC* variants, defined as having frequencies ≤ 1%, were identified. Population frequency data were obtained from the Franklin Genoox variant interpretation engine (https://franklin.genoox.com/clinical-db/home, accessed on 1 July 2024). From this cohort, individuals who had an additional pathogenic or likely pathogenic variant associated with FH or familial hypertriglyceridemia were also identified. The pathogenicity rating of these additional variants was found in the ClinVar database (https://www.ncbi.nlm.nih.gov/clinvar/, accessed on 1 July 2024) [23].

### 4.4. Variant Pathogenicity Classification

The pathogenicity of each variant was determined using the American College of Medical Genetics and Genomics (ACMG) Standards and Guidelines [24]. The pathogenicity rating determined by the Franklin Genoox variant interpretation engine was recorded (https://franklin.genoox.com/clinical-db/home, accessed on 1 July 2024) in addition to a manual classification [25]. The AI predictive algorithm VARITY was also used to predict the pathogenicity of each variant present in the cohort [18,19]. A VARITY score close to 1 indicates a high likelihood of pathogenicity [19].

### 4.5. Statistical Analysis

Microsoft Excel was used to analyze data and generate figures. The natural logarithm for TG values (lnTG) was used, given the non-normal distribution of this variable. Unpaired *t*-tests were performed to evaluate differences between groups. One-tailed *p*-values are reported for tests comparing lipids between *LIPC* and control cohorts. Two-tailed *p*-values are reported for all other tests. The Kruskal–Wallis test and Fisher’s exact test were used for very small numbers of patient subgroups. The *p*-value threshold of statistical significance was selected to be *p* ≤ 0.05.

## Figures and Tables

**Table 1:** Clinical and demographic information for simple heterozygotes with *LIPC* variants compared to controls.

	*LIPC* Simple Heterozygotes 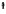	Control Subjects ^♦^
Number of patients/males	46/28	72/37
Age (years)	50 ± 14	44 ± 20
Body mass index (kg/m^2^)	28.4 ± 5.2	26.6 ± 4.9
Type 2 diabetes mellitus	6	0
History of atherosclerotic CVD	2	0
Current or former smoking history	12	n/a
No alcohol consumption	11	n/a
Total cholesterol (mmol/L, percentile)	6.46 ± 1.57 (75) *	5.11 ± 1.11(44)
LDL cholesterol (mmol/L, percentile)	3.75 ± 1.63 (62) *	3.19 ± 0.99 (38)
HDL cholesterol (mmol/L, percentile)	1.48 ± 0.71 (58)	1.34 ± 0.37 (53)
Triglycerides (mmol/L, percentile)	4.29 ± 4.99 (77) *	1.12 ± 0.48 (34)
Apolipoprotein B (g/L)	1.13 ± 0.32	1.07 ± 0.28

* *p* < 0.05 between groups. 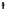 Body mass index (*n* = 45), smoking history (*n* = 37), alcohol consumption (*n* = 35), apolipoprotein B (*n* = 36). ^♦^ Body mass index (*n* = 71), apolipoprotein B (*n* = 53). Values shown are means ± standard deviation. Differences in the proportion of males, age, body mass index, and categorical values were not significant between groups.

**Table 2:** Clinical attributes of simple heterozygous patients with *LIPC* variants, subdivided by pathogenicity and VARITY score.

	Total Cholesterol (mmol/L)	LDL-C (mmol/L)	HDL-C (mmol/L)	Triglycerides (mmol/L)	Apo B (g/L)
Pathogenic (*n* = 3)	8.05	4.57	1.88	4.77	1.34
	*p* = 5.7 × 10^−5^	*p* = 0.011	*p* = 0.039	*p* = 0.0059 (log(TG))	0.031
Benign (*n* = 4)	5.98	2.75	1.46	4.16	1.20
	*p* = 0.159	*p* = 0.313	*p* = 0.385	*p* = 0.048 log(TG)	*p* = 0.25
VARITY (*n* = 10)	7.43	4.34	1.67	4.10	1.10
	*p* = 1.9 × 10^−3^	*p* = 0.061	*p* = 0.075	*p* = 6.0 × 10^−3^ log(TG)	*p* = 0.31

Abbreviations: low-density lipoprotein cholesterol (LDL-C), high-density lipoprotein cholesterol (HDL-C), apolipoprotein B (Apo B). Mean values are shown.

**Table 3:** Demographic and clinical characteristics of subjects with FH.

	Age	Sex	BMI * (kg/m^2^)	TC * (mmol/L)	Triglycerides (mmol/L)	HDL-C (mmol/L)	LDL-C (mmol/L)
Average ± SD (n = 135)	45 ± 14	45.2% M 54.8% F	27.6 ± 6.6	9.25 ± 1.96	1.62 ± 0.83	1.34 ± 0.62	7.20 ± 1.73

Abbreviations: body mass index (BMI), male (M), female (F), total cholesterol (TC), low-density lipoprotein cholesterol (LDL-C), high-density lipoprotein cholesterol (HDL-C), * BMI (*n* = 132), TC (*n* = 133). Values shown are means ± standard deviation.

## Data Availability

The data that support the findings of this study can be provided by the corresponding author, RAH, upon reasonable request.

## Supplementary Materials

The following supporting information can be downloaded at: https://www.mdpi.com/article/10.3390/ijms252111445/s1.
