# Supplementary material for: Phenotype in Individuals with Heterozygous Rare Variants in LIPC Encoding Hepatic Lipase
Source: Int J Mol Sci. 2024 Oct 24;25(21):11445. doi: 10.3390/ijms252111445 (PMC11546775; doi:10.3390/ijms252111445)
Supplement: Supplementary file 1 [file ijms-25-11445-s001.zip › ijms-3260441-supplementary.pdf]

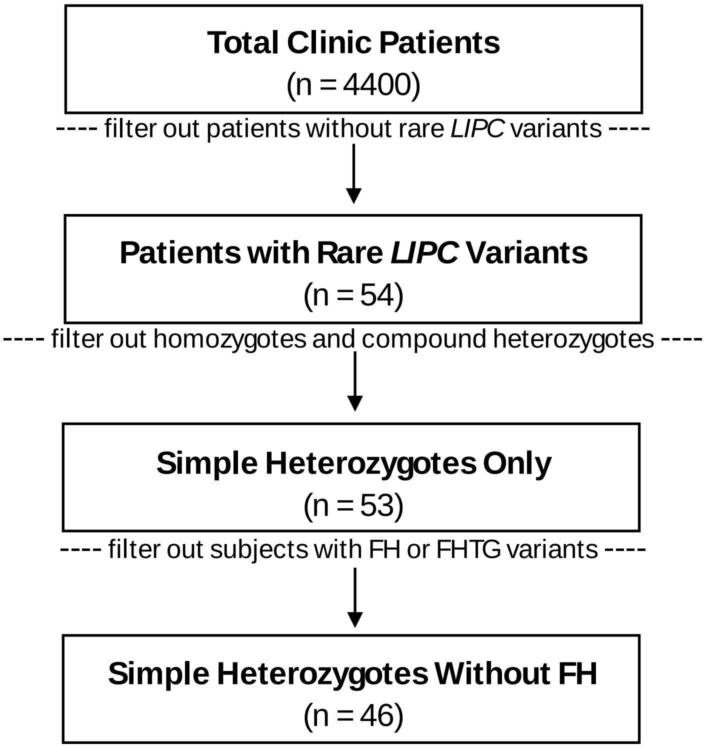

Figure S1. Flowchart outlining selection criteria for simple heterozygotes with *LIPC* variants. Out of 4400 total clinic patients who underwent next-generation DNA sequencing, 54 had heterozygous pathogenic variants in the *LIPC* gene. One patient, who was a compound heterozygote for 2 *LIPC* variants, was removed, leaving a subtotal of 53 simple heterozygotes for pathogenic *LIPC* variants. Next, patients with pathogenic variants in genes for familial hypercholesterolemia (FH) namely *LDLR*, *APOB*, *PCSK9* and *LDLRAP1* were filtered out. There were no individuals with pathogenic variants in genes for familial hypertriglyceridemia (FHTG) identified, leaving a final total of 46 simple heterozygotes for pathogenic *LIPC* variants, who were mainly studied here. Rare variants are defined as those with a population frequency ≤1%.

Table S1. *LIPC v*ariants present in the study cohort**.** Protein residue numbering includes the 22 amino acid pro-peptide sequence. Allele frequency was determined from gnomAD. Pathogenicity information was determined using ACMG guidelines and the Franklin Genoox variant interpretation engine. A bolded VARITY score indicates the variant is predicted by the algorithm to be pathogenic. The variant in the fourth row, H46P, is a novel variant.

Genomic location (GRCh37/ hg19)	cDNA change	Protein consequence	Allele frequency (gnomAD)	Snp138	Number of subjects	Pathogenicity rating	VARITY score
n/a	5UTR-exon1 del.	Deletion of Exon 1 (p.1-55 del.)	N/A	N/A	2	VUS	N/A
g.58724217	c.-15G>A	N/A	0.00003181	rs372805598	2	VUS	N/A
g.58724235	c.4G>T	p.D2Y	0.00001193	N/A	1	VUS	0.099
g.58830580	c.137A>C	p.H46P	N/A	N/A	2	VUS	0.076
g.58830639	c.196A>G	p.I66V	0.00009687	rs140029729	1	VUS	0.032
g.58830636	c.193C>T	p.R65X	0.000003976	rs369262181	2	Pathogenic	N/A

g.58830655	c.212C>T	p.T71M	0.00003229	N/A	1	VUS	0.228
g.58834015	c.306-320 del.	p.102-107 del.	N/A	N/A	1	VUS	N/A
g.58834131	c.421G>A	p.G141S	0.00003232	N/A	1	VUS	**0.902**
g.58837984	c.618C>A	p.S206R	0.00006457	rs199615899	1	VUS	0.179
g.58838051	c.685G>A	p.G229S	0.0000323	N/A	1	VUS	**0.952**
g.58838100	c.737-738insCG	p.N246fs	0.0002	N/A	2	VUS	N/A
g.58840535	c.815C>T	p.T272I	0.0007	rs148453176	2	VUS	0.212
g.58840586	c.866C>T	p.S289F	0.0011	rs121912502	3	Likely pathogenic	**0.934**
g.58840604	c.884C>T	p.T295M	0.00001194	N/A	1	VUS	0.035
g.58840751	c.1031G>C	p.R344P	0.000004735	N/A	1	VUS	**0.950**
g.58840718	c.998G>A	p.R333Q	0.00009694	rs200889722	1	VUS	0.017
g.58853075	c.1064G>A	p.Q355R	0.0006	rs140272400	2	Likely benign	0.580
g.58853166	c.1155A>T	p.K385N	0.00006461	N/A	1	VUS	0.071
g.58855760	c.1226A>C	p.D409A	0.0008	rs142036980	1	VUS	0.689
g.58855748	c.1214C>T	p.T405M	0.0055	rs113298164	8	Likely pathogenic	0.174
g.58855756	c.1222G>A	p.V408M	0.00003228	rs199654215	1	VUS	0.028
g.58855765	c.1231G>C	p.G411R	0.0003	N/A	2	VUS	**0.823**
g.58855772	c.1238T>C	p.L413P	0.0006	N/A	2	VUS	**0.937**
g.58860941	c.1415A>T	p.D472V	0.0005814	rs34596532	2	Likely benign	0.225
g.58860956	c.1430G>A	p.R477H	0.0002	rs148828229	2	VUS	0.016

Abbreviations: untranslated region (UTR), copy number variation (CNV), deletion (del), insertion (ins), stop-gain mutation (X), frameshift (fs), variant of uncertain significance (VUS), not available (N/A).
